# The capsular line reference, a new arthroscopic reference for posterior/anterior femoral tunnel positioning in anterior cruciate ligament reconstruction

**DOI:** 10.1186/s40634-018-0125-9

**Published:** 2018-03-27

**Authors:** Philippe Colombet, Alain Silvestre, Nicolas Bouguennec

**Affiliations:** Clinique du Sport de Bordeaux-Mérignac, 4 Rue Georges Negrevergne, 33700 Mérignac, France

**Keywords:** Anterior cruciate ligament, ACL reconstruction, Femoral tunnel positioning, Arthroscopic landmarks

## Abstract

**Background:**

Femoral malposition is the first cause for graft rupture during ACL reconstruction. Arthroscopic landmarks can be difficult to identify. So, landmark has to be found for reliable tunnel placement. A proximal-distal reference was described as “Apex reference” reported by Hart et al. but no posterior/anterior reference exists in the literature.

The purpose of this study was to do a 3D CT-scan assessment of the femoral tunnel positioning using the Capsular Line Reference (CLR) as a landmark for posterior/anterior placement in ACL reconstruction. We hypothesized the CLR could provide a precise and reliable antero/posterior femoral tunnel positioning less than 2 mm from the Bernard & Hertel posterior quarter.

**Methods:**

Seven cadaveric knee specimens with a mean age of 79.2 ± 11 years were used. Using standard approaches, the CLR was identified corresponding to a white line (the capsule) appearing at the posterior border of the femoral condyle after bony debridement of the medial and posterior part of the lateral femoral condyle. The center of the tunnel was marked. An inside-out technique with anteromedial drilling technique was performed using an 8-mm diameter reamer. The distal femurs were sawed and a CT-scan was done for each specimen to obtain 3-dimensional image reconstructions. These 3D reconstructions were analyzed to measure the position tunnel center on the posterior/anterior axis and the distance from the posterior/anterior quadrant according to the Bernard & Hertel method.

**Results:**

The mean position for the posterior/anterior axis was 27.0 ± 1.8% (25–28.9) with a median of 26.9%. The position from the first quarter of the Bernard & Hertel method was 0.9 ± 0.8 mm (0–1.8) with a median of 0.8 mm.

**Conclusion:**

The CLR is a reliable and reproducible arthroscopic landmark to place the femoral tunnel for ACL reconstruction in the anterior/posterior axis. Proximal/distal position depends on the choice of the surgeon to reproduce anteromedial or posterolateral fibers.

## Background

The most cited reason for residual instability and graft failure in arthroscopic anterior cruciate ligament (ACL) reconstruction remains technical errors including inappropriate graft fixation, graft impingement in the notch, incorrect femoral and tibial tunnel placement (Samitier et al., 2015; Wylie et al., 2017).

Appropriate tunnel placement is critical for the success of such surgeries, nevertheless, incorrect femoral tunnel placement still represents 80% of these technical errors (Samitier et al., 2015; Wylie et al., 2017; Whitehead, 2013; Trojani et al., 2011). Graft behavior is supported by anatomical, biomechanical and isometric conditions (Zavras et al., 2005). According to the most recent understanding of ACL anatomy (Smigielski et al., 2016; Smigielski et al., 2015; Sasaki et al., 2012), the graft must be placed anatomically on the lateral femoral condyle. Full anatomic ACL reconstruction remains actually a challenge, and the best we can achieve is a compromise between anatomy, biomechanics and isometry. In order to guide the surgeon in this choice, the concept of I.D.E.A.L femoral tunnel position has been proposed (Pearle et al., 2015). Arthroscopic ACL reconstruction is a technically demanding surgery and surgeons need easily recognizable bony landmarks in order to secure the tunnel placement. The tunnel center can be situated in two directions, Proximal and Distal and Posterior and Anterior positioning (Davis et al., 2016; Colombet et al., 2016). Many surgeons use the “classic clock face” centered in the notch, to place the tunnel center in proximal and distal position. This clock face technique is not precise enough and has been reported as lacking accuracy and precision for femoral tunnel placement (Azzam et al., 2011; Loriaut et al., 2017; Momaya et al., 2017). Some authors have shown that proper placement is related to the angle of knee flexion (Markolf et al., 2009), so the apex of posterior cartilage was recently reported as a better anatomical bony landmark for the proximal/distal position (Hart et al., 2015). Proximal/distal positioning depends on the ACL fibers the surgeon wishes to reconstruct: Antero-medial (AM) fibers are in the proximal part of the notch while Postero-lateral (PL) fibers are distal and both are anatomical.

The purpose of this cadaveric dissection study was to assess femoral tunnel positioning using the Capsular Line Reference (CLR) as a bony landmark for the posterior/anterior placement in arthroscopic ACL reconstruction. The CLR can be identified as a “white line” (corresponding to the capsule) appearing at the posterior border of the medial side of the lateral femoral condyle after bony debridement using a shaver. So it’s a posterior reference which can be always found and prevent to place the tunnel too anteriorly. In effect, the Posterior/Anterior position is much more crucial because a position which is too anterior constitutes a main reason for graft rupture (Samitier et al., 2015; Wylie et al., 2017). A femoral offset guide in different sizes (from 4 mm to 7 mm) has been proposed to control the Posterior/Anterior positioning (Tuca et al., 2016; Sekiya et al., 2016). It is hooked above the lateral femoral condyle but there is no visual arthroscopic landmark to ensure that the chosen target has been reached: the technique with offset guide has the disadvantage the position of the tunnel cannot be seen when the guide is in place whereas with the CLR technique, the position can be identified during all the procedure, regardless of the degree of flexion. Moreover, a simple synovial resection at the posterior part of the condyle is not enough to make appear the CLR and the posterior edge of the lateral notch wall is sometimes difficult to identify.

A 3D CT-scan of the specimen was used to precisely measure tunnel placement using the Bernard and Hertel (B&H) quadrant method (Bernard et al., 1997). Our hypothesis was that the CLR provides a precise and reliable antero/posterior femoral tunnel positioning less than 2 mm from the Bernard & Hertel posterior quarter (center of the ACL antero-medial fibers as described in the literature (Sullivan et al., 2015)).

## Method

### Cadaveric arthroscopy

**Fig. 1:**
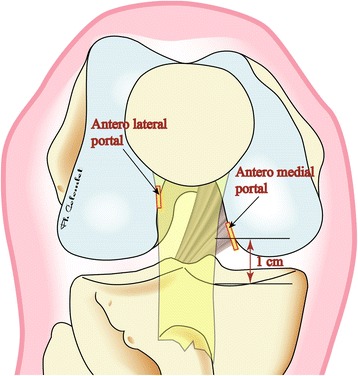
Picture showing the position of anteromedial and anterolateral portals. The anterolateral approach is proximal and close to the patellar tendon. The anteromedial approach is flush with the femoral medial condyle one centimeter above the tibial plateau

**Fig. 2:**
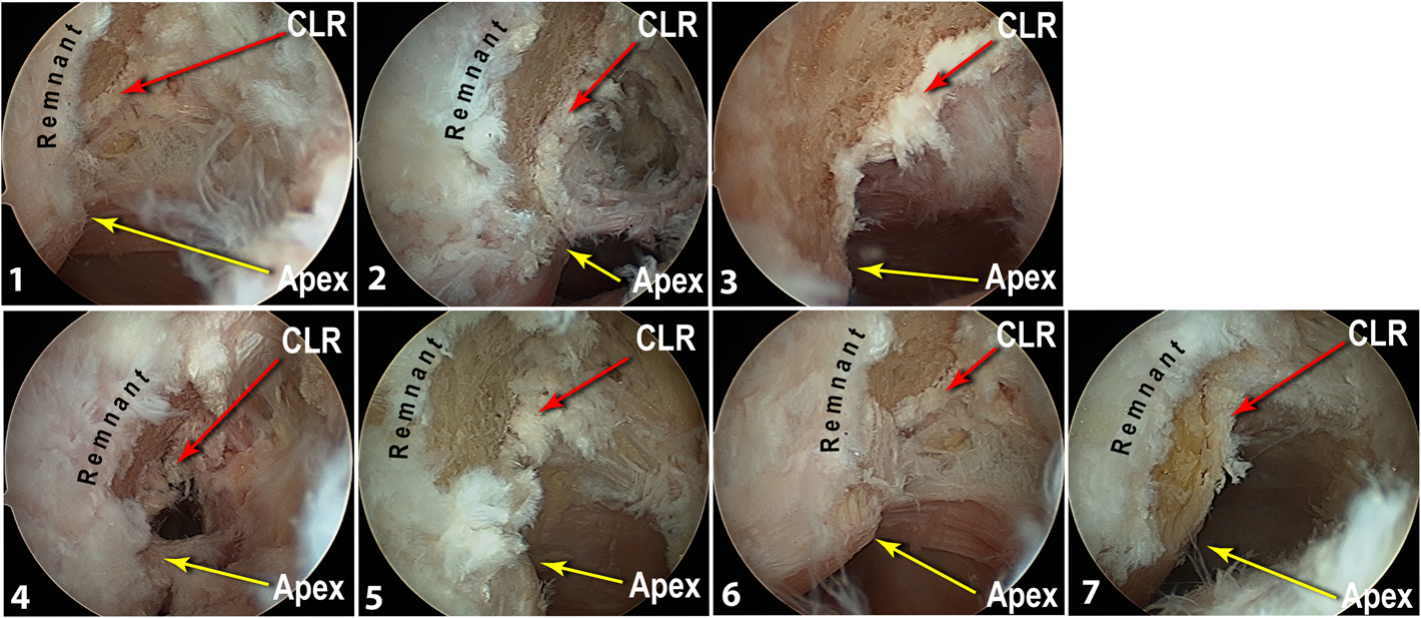
Arthroscopic views of the right knees (through anterolateral portal) medial part of the lateral femoral condyle for the seven specimens after debridement. For each one, Capsular Line Reference (red arrow), apex of the posterior cartilage (yellow arrow) and remnant fibers of the ACL are shown

Seven deep frozen cadaveric knees were used (mean age 78.5 ± 9 years) and thawed at room temperature 24 h before the experiment. Consent was given during the lifetime for the use of specimen for research. They were all right knees. The specimen inclusion criteria were no scars from previous surgery, no significant osteoarthritis with only grade I or II chondral lesions (ICRS Classification). The femur was fixed on the table with a metallic holder. We used an anterolateral arthroscopic portal and an anteromedial instrumental portal. The anterolateral portal was placed at the corner of the patellar tendon lateral edge and the patella. The anteromedial portal was situated in the notch’s medial wall alignment, one centimeter above the medial joint line (Fig. 1). A classic 30° angle scope was used and we took a notch picture before removing the ACL with a shaver (Formula Shaver Striker cutter blade aggressive plus 5 mm) introduced by the anteromedial portal. Next, the shaver blade was used as a burr (6000 rpm) in order to clean out the soft tissue and a little of bone from the posterior part of the lateral notch wall. After that trick, a “white line” (corresponding to the capsule) appeared at the posterior border of the medial side of the lateral femoral condyle. This is the arthroscopic landmark identified as the Capsular Line Reference (CLR) (Fig. 2). The CLR is so placed at the posterior edge of the condyle, below the roof of the notch but appeared only after bony debridement of the medial part of the condyle.

### Landmark

**Fig. 3:**
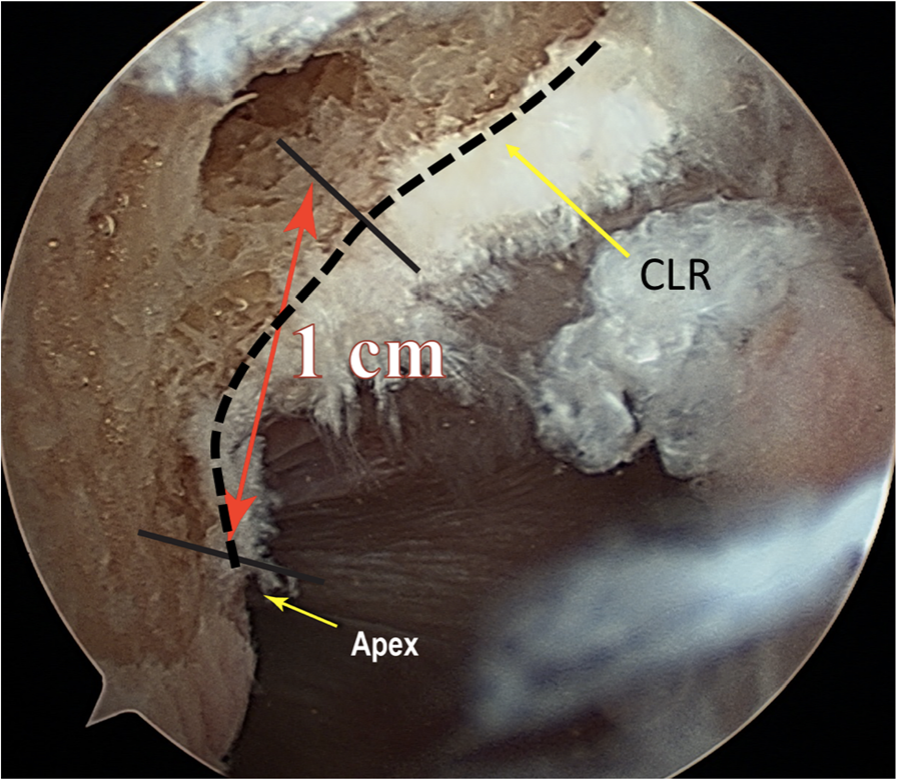
Arthroscopic view of the lateral notch through the anterolateral approach of a right knee showing the position of the center of the tunnel: close to the CLR and one centimeter higher (anteriorly) relative to the apex of the posterior cartilage. The CLR is marked

**Fig. 4:**
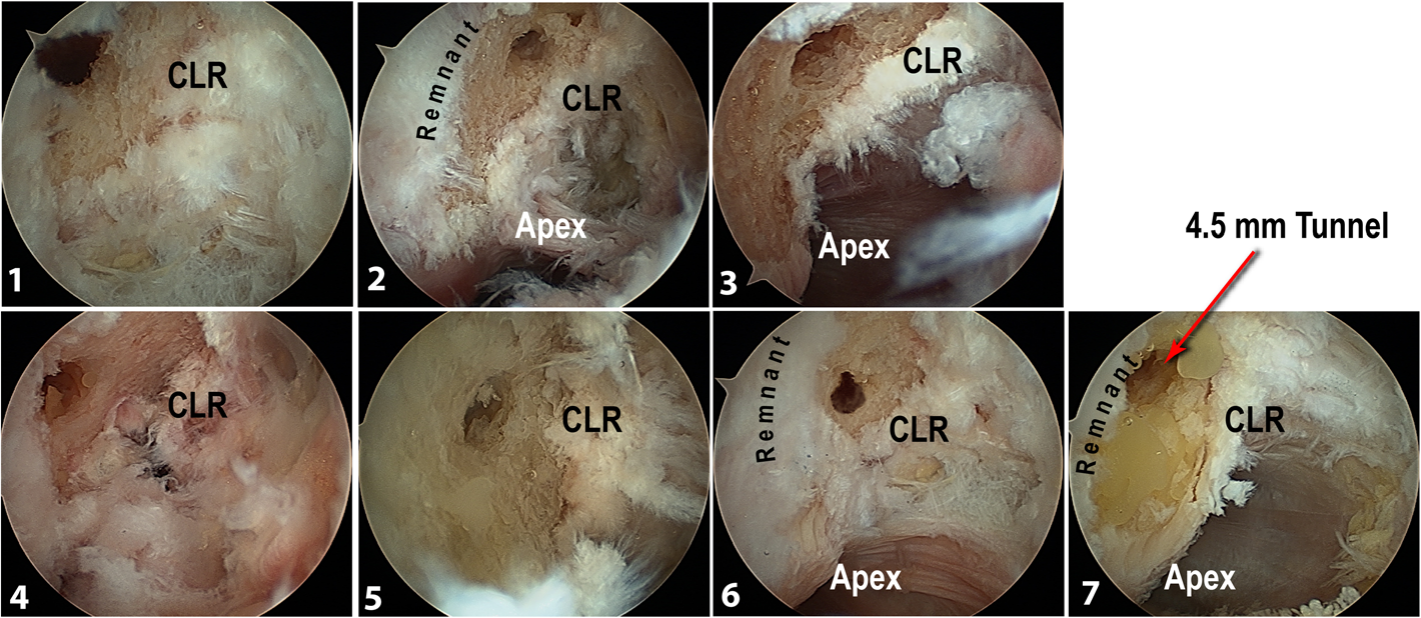
Arthroscopic views (through an anterolateral approach of the right knees) of the medial part of the lateral femoral condyle with tunnel center location on the seven specimens

The CLR was used for the anterior-posterior position of the tunnel. The center of our femoral tunnel was placed in front of the CLR and at the level of Antero Medial (AM) native ACL direct fiber attachment: using the CLR as the posterior reference, the aim was to be as posterior as possible. As we used after a 7 mm diameter drill, we placed the center of the tunnel at 5 mm anteriorly relative to the CLR. The proximal and distal positions in the notch were controlled using the Apex of the Posterior Cartilage (Hart et al., 2015) at the posterior part of the lateral condyle. We placed the drill tip 10 mm from this apex (Fig. 3). The drill was oriented as much as possible in an anterior and proximal direction after positioning the knee at 120° of flexion, the limitation was given by contact of the drill with the medial condyle. We used a 4.5 mm drill equipped with a sharp tip. This tip is used to control the position of the drill and keep the drill in the chosen position while the knee is flexed from 90° to 120°. A 4.5 mm epiphysis tunnel was drilled through and the lateral femoral cortex was perforated using an anteromedial drilling technique and flexion of the knee at 120°. At this point, arthroscopic control was done and tunnel placement was considered as acceptable when there remained 1 mm between the posterior part of the tunnel and the CLR (Fig. 4). Then, a k-wire was introduced in the tunnel while the knee was still bent to 120° of flexion. We used a 7 mm diameter drill to create a 25 mm length socket.

### Cadaveric dissection and CT scan

**Fig. 5:**
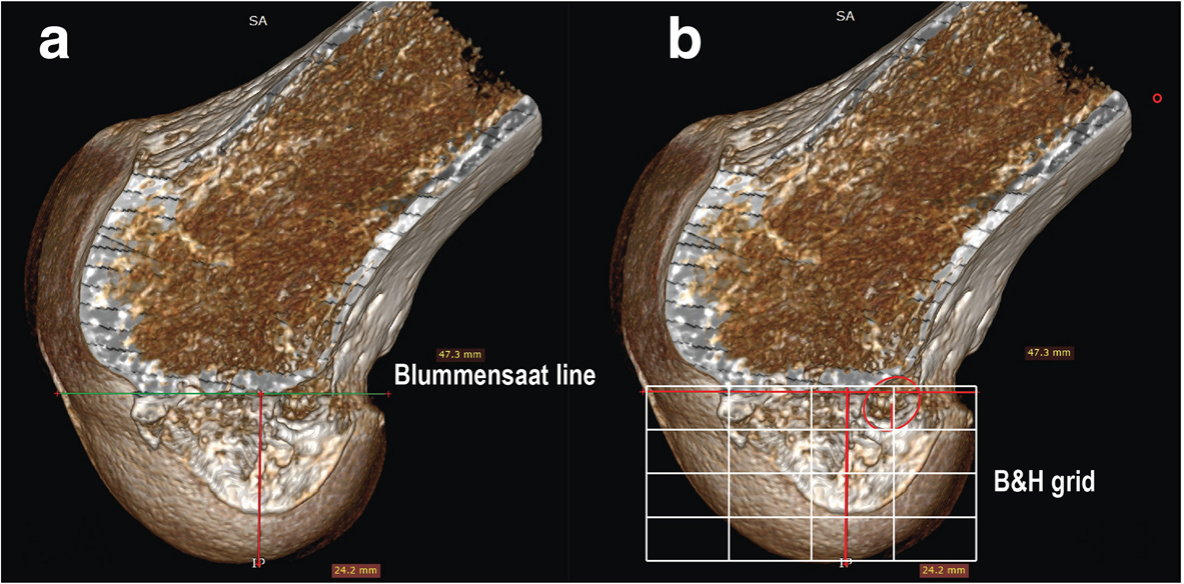
**a** 3D CT-scan reconstruction of the lateral femoral condyle. Length and width of the two axes of the construction of Bernard & Hertel quadrant method are measured. **b** Example of tunnel center position measures using Bernard & Hertel quadrant method on the 3D CT-scan reconstruction

All the soft tissue was removed from the femoral extremity and pictures were taken, then, the femur was inserted in a plastic bag. The specimens were placed one by one in a CT scan (General Electric Optima CT580W 16fps) and computerized tomography was done for each femur. A DICOM file was created for each specimen and we used RadiAnt DICOM viewer® (Medixant 1.9.16 Poznan Poland) Software for picture analysis. The 3D volume rendering function was used. The 3D femur was rotated along a vertical axis in order to superimpose both condyles. Next, the selection was rotated by 90 degrees and a scalpel tool was used to cut the distal femoral epiphysis in the middle of the notch before the medial condyle was removed. We rotated back by 90° to return to a medial view of the lateral condyle. We used the length tool to measure the Bernard & Hertel quadrant sides (Bernard et al., 1997) (length and width) (Fig. 5a). This picture was exported as a JPEG file in Adobe illustrator CS6.

### Imaging measurements

A scaled illustration of the femoral footprint was produced using Adobe Illustrator® C26 graphics software. The tunnel aperture was drawn and its center located (Fig. 5b). The Bernard & Hertel quadrant was drawn and its dimensions measured (length. width) in Adobe illustrator were related to the real distance reported in Radian DICOM viewer picture. We measured the distance in millimeters (mm) from the posterior part of the quadrant to the tunnel center and was recorded as a percentage with the quadrant’s Blumensaat line. Then, we measured the distance from the tunnel center to the first posterior quarter of the Bernard & Hertel quadrant which is the center of the AM direct fibers of the native ACL (Bernard et al., 1997; Johnson et al., 2015; Jenny et al., 2011; Kawakami et al., 2012; Lee et al., 2007; Li et al., 2012; Youm et al., 2013). The first posterior quarter was considered as the position to target and the aim was to bas as close as possible from the first quarter. Statistical Analysis: Descriptive statistics (mean, median and standard Deviation) were used to describe the anatomical relationships measured in this study.

## Results

**Fig. 6:**
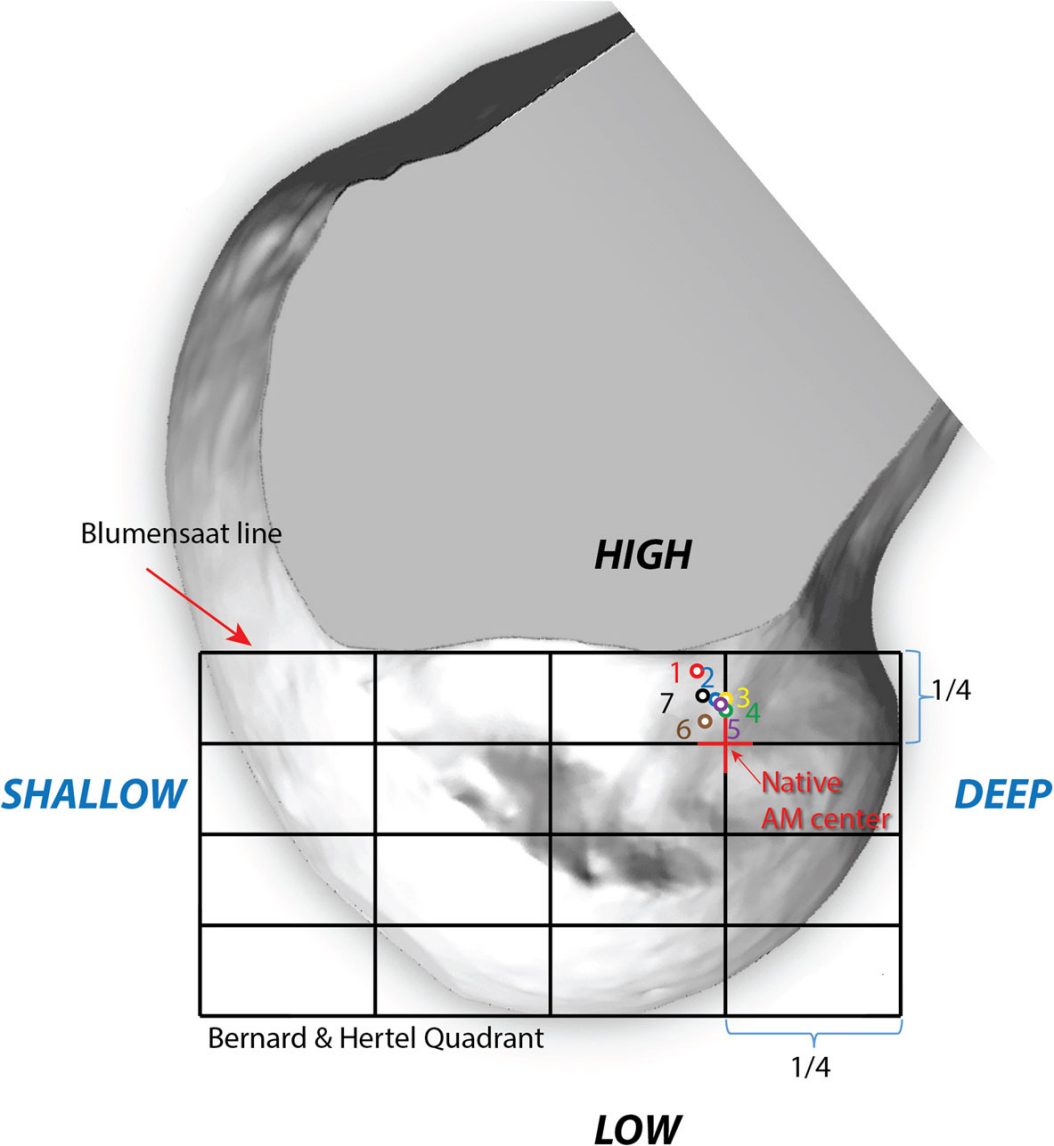
Right femur: mapping each tunnel center measures (1 to 7). The Bernard & Hertel quadrant is drawn in the black grid. The center of the AM direct fibers of the ACL is placed at the Red Cross situated one quarter from the top and one quarter from the posterior part of the B&H grid. The upper part of the grid is the Blumensaat line

Analysis was possible for the seven specimens. Regarding the Bernard & Hertel quadrant edges, all the specimens were homogenous. The Blumensaat line mean length was 45.0 mm ± 1.9 (42.5 to 47.3 mm) and the posterior/anterior mean length was 22.1 mm ± 2.3 (18.9 to 25 mm). The distance of the tunnel center from the Bernard & Hertel posterior quarter line was 0.9 ± 0.8 mm (0–1.8) with a median of 0.8 mm. All the centers were located on the anterior side of this line (Fig. 6). When we considered the femoral tunnel center distance from the posterior edge of B&H quadrant as a percentage, the average mean position for the posterior/anterior axis was 27.0 ± 1.8% (25–28.9). The median was 26.9%. Results are summarized in Table 1.

**Table 1:** Results for the seven specimens with two analyses: The distance of the tunnel center from the Bernard & Hertel (B&H) deep quarter line according to the quadrant method

	B&H dimensions		Deep/Shallow measurement from the deep			Distance from the AM target
	High/Low length	Deep/Shallow length	Native AM center	Tunnel center		
	mm	mm	mm	mm	%	mm
Specimen 1	22,7	45,2	11,3	13,1	28,9%	**1,8**
Specimen 2	23,7	43,4	10,85	11,7	26,9%	**0,8**
Specimen 3	19,5	45,8	11,45	11,5	25,0%	**0,0**
Specimen 4	24,20	47,30	11,825	11,8	25,0%	**0,0**
Specimen 5	25	47,5	11,875	12,1	25,5%	**0,2**
Specimen 6	18,9	42,5	10,625	12,3	28,9%	**1,7**
Specimen 7	21	43,6	10,9	12,5	28,6%	**1,6**
**Mean**	**22,1**	**45,0**	**11,3**	**12,1**	**27,0%**	**0,9**
S Deviation	2,38	1,96	0,49	0,55	1,8%	0,80
Median	22,7	45,2	11,3	12,1	26,9%	0,8

## Discussion

The main result of this study is that the Capsular Line Reference seems to be a reliable and reproducible reference for the positioning of the femoral tunnel in arthroscopic ACL reconstruction. Our hypothesis was validated with a mean distance from the posterior edge of B&H quadrant at 0.9 ± 0.8 mm (0–1.8).

Initially described as a radiological measuring method in 1996 in a German journal (Bernard & Hertel, 1996) and popularized since 1997, the Bernard & Hertel quadrant method is the most widely used in the literature for analyzing the position of the femoral tunnel on a lateral view (Bernard et al., 1997), (Hwang et al., 2012). This method was validated by Kim et al. for CT-scan analysis (Kim et al., 2016) and its reliability was confirmed by Lertwanich et al. (Lertwanich et al., 2011). We chose CT-scan analysis because it is more precise as it allows for accurate orientation of the medial part of the lateral condyle and offers the possibility of having measurements in millimeters without parallax or length approximation.

In our study, average mean position of the femoral tunnel center was at 27.0 ± 1.8% and the median was 26.9% for posterior/anterior position according to the Bernard & Hertel method. This result corresponds to the literature. Parkar et al. (Parkar et al., 2016) recently did a systematic review of imaging and cadaveric analysis of the position of tunnels in ACL reconstruction and reported a mean of 28.6% and a median of 26.3% for the posterior/anterior position for single bundle reconstruction. Results were comprised between 23.5 and 37.3%. For double-bundle reconstructions, the anteromedial bundle was comprised between 15 and 33.9%. We also found a small standard deviation (27.0 ± 1.8%) showing the good reproducibility of the CLR technique.

Numerous techniques describe landmarks and references to correctly place the femoral tunnel center, including: clock face method, bony landmarks, ACL remnant, Computer-Assisted Surgery (CAS) and radiographic method (Davis et al., 2016). The clock face method is not precise because it depends on the position of the knee and cannot be proposed as a reliable method (Davis et al., 2016). In 2012, Piefer et al. (Piefer et al., 2012) summarized the different bony landmarks (lateral intercondylar ridge, “back” of femur, notch roof, “over-the-top” position, posterior cortical border of femoral diaphysis). They specified that ACL insertion is located in an area below the notch roof and posteriorly to the lateral intercondylar ridge but this corresponds to a large area. Pansard et al. also proposed to only use bony landmarks to place the tunnel because they found no difference between two types of drilling techniques (Pansard et al., 2015). But as written by Steckel et al., visualization of the footprint varies with degrees of flexion, and so, a slight variation could modify the position of the tunnel even though bone is seen (Steckel et al., 2010). In addition, bony landmarks such as the lateral intercondylar ridge can be difficult to identify. Another landmark described is the stump of the ACL. It can be used in addition to bony landmarks but the size of the footprint is an oval with a length ranging from 12.9 to 18.4 mm and a width between 4.7 and 9.3 mm (Piefer et al., 2012) so it cannot be fully covered and a choice has to be done to place the tunnel inside the stump. Radiographic and CAS are time consuming or expensive and difficult to use in a daily practice everywhere. Davis et al. (Davis et al., 2016) published a technique to place the femoral tunnel by measuring the height and the depth of the lateral notch but this implies parallax approximation and does not take into account variations in patient anatomy. Hart et al. (Hart et al., 2015) proposed the Apex of Posterior Cartilage (ADC) as a reference. This method aims to place the tunnel at the center of the footprint by measuring a fixed length from the ADC. Once again, this technique ignores variations in anatomy and using only fixed measured distance does not seem to be optimal. Furthermore, the center of the footprint is not the position we target with the CLR technique, we aim to place the center of the tunnel in the middle of the anteromedial fibers of the native ACL. The position of these anteromedial fibers corresponds to the first posterior/anterior quarter line of the Bernard & Hertel quadrant (Bernard et al., 1997; Johnson et al., 2015; Jenny et al., 2011; Kawakami et al., 2012; Lee et al., 2007; Li et al., 2012; Youm et al., 2013). We found a distance of 0.9 ± 0.8 mm from this line showing the CLR as a precise posterior/anterior landmark to place the tunnel at insertion sites of anteromedial fibers. Ahn et al. measured the distance from a reference point using the quadrant method, but the results were given as a percentage and not in millimeters (Ahn et al., 2013).

We chose the insertion site of anteromedial fibers because Pearle et al. advocated that the aim of ACL reconstruction could not be just to try to fill the footprint and centralize the center of the tunnel and so that a compromise should be an option (Pearle et al., 2015). Although the anatomy of the footprint varies from patient to patient, there is some consistent data in the anatomy of the ACL which structure was recently described as ribbon like (Smigielski et al., 2016). The anterior margin of the direct fibers is the intercondylar bony ridge and there is a fan-like extension posteriorly close to the cartilage, and in continuity with the capsule attachment on the lateral condyle (Mochizuki et al., 2014). Iwahashi reported an average width of the insertion of 8 mm. Given its direction, the graft does not fill up the whole tunnel aperture but occupies the anterior part of it (Segawa et al., 2003). When the tunnel is placed behind the intercondylar bony ridge and anteriorly to the capsule, the graft is situated in the native anterior direct fiber location. With the CLR technique, this criterion is fulfilled because the reference is the capsule; the tunnel is placed behind the interconyldar ridge. Moreover, the proximal and anterior part of the native ACL insertion (called anteromedial fibers) is the most isometric location (Simmons et al., 2003), these fibers are subjected to small elongations during flexion extension of the knee and thus correspond to a placement with low tension applied on the graft. So, with the CLR technique, the tunnel is placed at the proximal and anterior part of the femoral footprint, which corresponds to the I.D.E.A.L. position for the center of the tunnel, as recommended by Pearle et al. (Pearle et al., 2015). So the CLR is a posterior reference that can be found in all patients and allows to be as posterior as possible. It’s necessary to do the bony debridement to make it appear at the posterior edge of the condyle.

There are, however, some limitations. We studied seven specimens but this number corresponds to the literature (Hart et al., 2015), (Johnson et al., 2015), (Musahl et al., 2003) and we found a small standard deviation showing the reliability of the technique. One surgeon performed the technique for all the specimens but analysis was done by an independent observer. Our study is a cadaveric analysis but we applied the exact same operative conditions with the same instruments so we could expect similar results in human surgery. A prospective clinical study with the CLR technique is on the way.

## Conclusion

This study shows that the CLR reference appears to be a reliable and precise technique for femoral tunnel placement during an ACL reconstruction. This arthroscopic reference could substantially help in posterior/anterior femoral tunnel positioning.

## References

[CR1] Ahn JH, Lee YS, Ko TS, Shin JY (2016) Accuracy and reproducibility of the femoral tunnel with different viewing techniques in the ACL reconstruction. Orthopedics 39(6):e1085-e1091. 10.3928/01477447-20160719-08 10.3928/01477447-20160719-08 27459141

[CR2] Azzam MG, Lenarz CJ, Farrow LD, Israel HA, Kieffer DA, Kaar SG (2011). Inter- and intraobserver reliability of the clock face representation as used to describe the femoral intercondylar notch. Knee Surg Sports Traumatol Arthrosc.

[CR3] Bernard M, Hertel P (1996). Intraoperative and postoperative insertion control of anterior cruciate ligament-plasty. A radiologic measuring method (quadrant method). Unfallchirurg.

[CR4] Bernard M, Hertel P, Hornung H, Cierpinski T (1997). Femoral insertion of the ACL. Radiographic quadrant method. Am J Knee Surg.

[CR5] Colombet P, Graveleau N, Jambou S (2016). Incorporation of hamstring grafts within the Tibial tunnel after anterior cruciate ligament reconstruction: magnetic resonance imaging of suspensory fixation versus interference screws. Am J Sports Med.

[CR6] Davis AD, Manaqibwala MI, Brown CH, Steiner ME (2016). Height and depth guidelines for anatomic femoral tunnels in anterior cruciate ligament reconstruction: a cadaveric study. Arthroscopy.

[CR7] Hart A, Han Y, Martineau PA (2015). The apex of the deep cartilage: a landmark and new technique to help identify femoral tunnel placement in anterior cruciate ligament reconstruction. Arthroscopy.

[CR8] Hwang MD, Piefer JW, Lubowitz JH (2012). Anterior cruciate ligament tibial footprint anatomy: systematic review of the 21st century literature. Arthroscopy.

[CR9] Jenny JY, Ciobanu E, Clavert P, Jaeger JH, Kahn JL, Kempf JF (2011). Anatomic attachment of the ACL. Comparison between radiological and CT analysis. Knee Surg Sports Traumatol Arthrosc.

[CR10] Johnson JS, Smith SD, LaPrade CM, Turnbull TL, LaPrade RF, Wijdicks CA (2015). A biomechanical comparison of femoral cortical suspension devices for soft tissue anterior cruciate ligament reconstruction under high loads. Am J Sports Med.

[CR11] Kawakami Y, Hiranaka T, Matsumoto T, Hida Y, Fukui T, Uemoto H, Doita M, Tsuji M, Kurosaka M, Kuroda R (2012). The accuracy of bone tunnel position using fluoroscopic-based navigation system in anterior cruciate ligament reconstruction. Knee Surg Sports Traumatol Arthrosc.

[CR12] Kim DH, Lim WB, Cho SW, Lim CW, Jo S (2016) Reliability of 3-Dimensional Computed Tomography for Application of the Bernard Quadrant Method in Femoral Tunnel Position Evaluation After Anatomic Anterior Cruciate Ligament Reconstruction. Arthroscopy. 10.1016/j.arthro.2016.01.043 10.1016/j.arthro.2016.01.043 27090722

[CR13] Lee MC, Seong SC, Lee S, Chang CB, Park YK, Jo H, Kim CH (2007). Vertical femoral tunnel placement results in rotational knee laxity after anterior cruciate ligament reconstruction. Arthroscopy.

[CR14] Lertwanich P, Martins CA, Asai S, Ingham SJ, Smolinski P, Fu FH (2011). Anterior cruciate ligament tunnel position measurement reliability on 3-dimensional reconstructed computed tomography. Arthroscopy.

[CR15] Li H, Yao Z, Jiang J, Hua Y, Chen J, Li Y, Gao K, Chen S (2012). Biologic failure of a ligament advanced reinforcement system artificial ligament in anterior cruciate ligament reconstruction: a report of serious knee synovitis. Arthroscopy.

[CR16] Loriaut P, Moreau PE, Boyer P (2017). Arthroscopic treatment of displaced tibial eminence fractures using a suspensory fixation. Indian J Orthop.

[CR17] Markolf KL, Park S, Jackson SR, McAllister DR (2009). Anterior-posterior and rotatory stability of single and double-bundle anterior cruciate ligament reconstructions. J Bone Joint Surg Am.

[CR18] Mochizuki T, Fujishiro H, Nimura A, Mahakkanukrauh P, Yasuda K, Muneta T, Akita K (2014). Anatomic and histologic analysis of the mid-substance and fan-like extension fibres of the anterior cruciate ligament during knee motion, with special reference to the femoral attachment. Knee Surg Sports Traumatol Arthrosc.

[CR19] Momaya AM, Read C, Steirer M, Estes R (2017) Outcomes after arthroscopic fixation of tibial eminence fractures with bioabsorbable nails in skeletally immature patients. J Pediatr Orthop B. 10.1097/BPB.0000000000000459 10.1097/BPB.0000000000000459 28368929

[CR20] Musahl V, Burkart A, Debski RE, Van Scyoc A, Fu FH, Woo SL (2003) Anterior cruciate ligament tunnel placement: Comparison of insertion site anatomy with the guidelines of a computer-assisted surgical system. Arthroscopy. 19 (2):154–160. doi:10.1053/jars.2003.50001 10.1053/jars.2003.50001 12579148

[CR21] Pansard E, Klouche S, Vardi G, Greeff E, Hardy P, Ferguson M (2015). How accurate are anatomic landmarks for femoral tunnel positioning in anterior cruciate ligament reconstruction? An in vivo imaging analysis comparing both anteromedial portal and outside-in techniques. Arthroscopy.

[CR22] Parkar AP, Adriaensen ME, Vindfeld S, Solheim E (2016) The anatomic centers of the femoral and Tibial insertions of the anterior cruciate ligament: a systematic review of imaging and cadaveric studies reporting normal center locations. Am J Sports Med. 10.1177/0363546516673984 10.1177/0363546516673984 27899355

[CR23] Pearle AD, McAllister D, Howell SM (2015). Rationale for strategic graft placement in anterior cruciate ligament reconstruction: I.D.E.A.L. femoral tunnel position. Am J Orthop (Belle Mead NJ).

[CR24] Piefer JW, Pflugner TR, Hwang MD, Lubowitz JH (2012). Anterior cruciate ligament femoral footprint anatomy: systematic review of the 21st century literature. Arthroscopy.

[CR25] Samitier G, Marcano AI, Alentorn-Geli E, Cugat R, Farmer KW, Moser MW (2015). Failure of anterior cruciate ligament reconstruction. Arch Bone Jt Surg.

[CR26] Sasaki N, Ishibashi Y, Tsuda E, Yamamoto Y, Maeda S, Mizukami H, Toh S, Yagihashi S, Tonosaki Y (2012). The femoral insertion of the anterior cruciate ligament: discrepancy between macroscopic and histological observations. Arthroscopy.

[CR27] Segawa H, Koga Y, Omori G, Sakamoto M, Hara T (2003). Influence of the femoral tunnel location and angle on the contact pressure in the femoral tunnel in anterior cruciate ligament reconstruction. Am J Sports Med.

[CR28] Sekiya H, Takatoku K, Kimura A, Kanaya Y, Fukushima T, Takeshita K (2016). Arthroscopic fixation with EndoButton for tibial eminence fractures visualised through a proximal superomedial portal: a surgical technique. J Orthop Surg (Hong Kong).

[CR29] Simmons R, Howell SM, Hull ML (2003). Effect of the angle of the femoral and tibial tunnels in the coronal plane and incremental excision of the posterior cruciate ligament on tension of an anterior cruciate ligament graft: an in vitro study. J Bone Joint Surg Am.

[CR30] Smigielski R, Zdanowicz U, Drwiega M, Ciszek B, Ciszkowska-Lyson B, Siebold R (2015). Ribbon like appearance of the midsubstance fibres of the anterior cruciate ligament close to its femoral insertion site: a cadaveric study including 111 knees. Knee Surg Sports Traumatol Arthrosc.

[CR31] Smigielski R, Zdanowicz U, Drwiega M, Ciszek B, Williams A (2016). The anatomy of the anterior cruciate ligament and its relevance to the technique of reconstruction. Bone Joint J.

[CR32] Steckel H, Musahl V, Fu FH (2010). The femoral insertions of the anteromedial and posterolateral bundles of the anterior cruciate ligament: a radiographic evaluation. Knee Surg Sports Traumatol Arthrosc.

[CR33] Sullivan JP, Cook S, Gao Y, Wolf BR (2015). Radiographic anatomy of the native anterior cruciate ligament: a systematic review. HSS J.

[CR34] Trojani C, Sbihi A, Djian P, Potel JF, Hulet C, Jouve F, Bussiere C, Ehkirch FP, Burdin G, Dubrana F, Beaufils P, Franceschi JP, Chassaing V, Colombet P, Neyret P (2011). Causes for failure of ACL reconstruction and influence of meniscectomies after revision. Knee Surg Sports Traumatol Arthrosc.

[CR35] Tuca M, Luderowski E, Rodeo S (2016). Meniscal transplant in children. Curr Opin Pediatr.

[CR36] Whitehead TS (2013). Failure of anterior cruciate ligament reconstruction. Clin Sports Med.

[CR37] Wylie JD, Marchand LS, Burks RT (2017). Etiologic factors that lead to failure after primary anterior cruciate ligament surgery. Clin Sports Med.

[CR38] Youm YS, Cho SD, Eo J, Lee KJ, Jung KH, Cha JR (2013). 3D CT analysis of femoral and tibial tunnel positions after modified transtibial single bundle ACL reconstruction with varus and internal rotation of the tibia. Knee.

[CR39] Zavras TD, Race A, Amis AA (2005). The effect of femoral attachment location on anterior cruciate ligament reconstruction: graft tension patterns and restoration of normal anterior-posterior laxity patterns. Knee surgery, sports traumatology, arthroscopy : official journal of the ESSKA.

